# Unequal effects of the COVID-19 epidemic on employment: Differences by immigrant status and race/ethnicity

**DOI:** 10.1371/journal.pone.0277005

**Published:** 2022-11-15

**Authors:** Kristin Tianqi Liao, Andrés Villarreal

**Affiliations:** University of California, Los Angeles, California, United States of America; Universidade Federal de Minas Gerais, BRAZIL

## Abstract

The COVID-19 epidemic resulted in a dramatic contraction in employment in the U.S., but the effects of this contraction have been unevenly distributed. We examine differences in employment among foreign- and native-born workers by race/ethnicity during the course of the epidemic. We test individual fixed-effects models based on data from the monthly CPS panel from January 2020 to December 2021 adjusting for seasonality. Immigrant men and women experienced greater declines in employment than non-immigrants of the same race/ethnicity when both compared to native-born Whites, but their disadvantage were limited to the initial months of the epidemic. Ethnoracial and immigrant status disparities were substantially reduced by the fall of 2020, except for Hispanic immigrant men and women, who still experienced substantial employment gaps with their native-born White counterparts. Differences in family characteristics account for Hispanic immigrant women’s lower employment rates during the epidemic but do not appear to account for differences between Black and Asian women and native-born Whites. Observed disparities in employment by race/ethnicity and immigrant status cannot be fully explained by differences in education, the concentration of minority and immigrant workers in industries and occupations that suffered steeper employment declines, or regional differences in the intensity of the epidemic.

## Introduction

The COVID-19 epidemic has greatly disrupted U.S. labor markets. Government policies intended to reduce the spread of the virus, including the shutdown of non-essential businesses, stay-at-home orders, and school closures reduced the supply and demand of labor. Fear of infection also led many individuals to stay at home or substantially curtail their economic activity. In the months following the onset of the epidemic in March 2020, the U.S. unemployment rate reached its highest level since the 1930s [1]. Many other workers withdrew from the labor force altogether [2]. Given the detrimental effects that even temporary job loss and unemployment may have on individuals’ well-being [3, 4], the economic consequences of the epidemic may further contribute to its long-term toll on human lives.

However, just as infection rates have varied significantly across ethnoracial groups [5, 6], so too have the labor market effects of the epidemic. Early research on the economic impact of COVID-19 shows that Hispanic men experienced the greatest increase in unemployment of any ethnoracial group and were more likely to withdraw from the labor force [7, 8]. The unemployment rate of African-American men also increased more than that of White men although the disparity was not as large as in previous recessions [7]. Asian men without a college education were likewise negatively affected during the early stages of the epidemic-induced recession [9].

Comparatively less is known about how the COVID-19 epidemic affected immigrant workers. Immigrant men are often employed in more precarious conditions and are therefore the first to be laid off during economic downturns [10–12]. Evidence indeed suggests that immigrant men and women had lower employment rates than their native-born counterparts in the early part of the epidemic [13]. Yet research on the employment of immigrants during this period has not examined differences by race and ethnicity. Given the disproportionate share of immigrants who are racial or ethnic minorities, overall differences in employment by immigrant status may partly reflect ethnoracial disparities.

Because the contraction in economic activity as a result of the epidemic differed in important respects from that of previous recessions, we may expect it to have had a different impact on employment patterns across sociodemographic groups. First, the COVID-19 epidemic negatively affected a different set of industries and occupations than in past recessions. For example, the leisure and hospitality industries, which generally employ a larger share of African Americans and Hispanics [14], were among the hardest hit in the COVID-driven recession [15]. Job losses in the manufacturing sector, which employ a smaller share of minority workers, were not as severe as in past recessions [16]. These differences suggest a larger impact of the COVID-19 recession on existing ethnoracial disparities in employment. On the other hand, the construction industry, which suffered a particularly large contraction during the Great Recession of 2008 and employs a disproportionate number of Hispanic and immigrant workers, was relatively spared during the COVID-19 recession since it was considered essential [17].

Second, differences in the effect of COVID-19 restrictions on employment by occupations may also account for ethnoracial disparities. Non-White and foreign-born workers with lower levels of education are less likely to work in occupations whose tasks can be performed remotely [13, 18, 19], and may therefore have been at higher risk of being laid off or having their hours reduced.

Finally, regional differences in the impact of the COVID-19 epidemic and in government policies to reduce the transmission of the virus may also affect the overall rates of employment by race/ethnicity and immigrant status. For example, Hispanics, Asians and first-generation immigrants are concentrated in states such as California, Texas, Florida and New York [20]. These states were among those with the highest early infection rates. States also differed in the timing of policies that restrict economic activity [21]. The concentration of minority and immigrant workers in states that were early adopters of shutdowns and school closures could also affect ethnoracial and immigrant status disparities.

In this study, we examine the intersecting effect of immigrant status and race/ethnicity on employment differences during the COVID-19 epidemic. We make several contributions to the literature. First, we examine the interaction between race/ethnicity and immigrant status in employment outcomes. Previous research on the effects of the COVID-19 epidemic has focused on differences in employment by either race/ethnicity or immigrant status separately [13, 22, 23]. However, as we demonstrate below, such a strategy risks confounding the effects of the epidemic on immigrant workers with those on ethnoracial minorities since a disproportionate share of immigrant workers are non-White.

Second, our study tests three potential explanations for employment disparities between native and foreign-born workers of different race and ethnicity. Specifically, we consider whether the disparities in employment are a result of differences in educational attainment, job characteristics and the concentration of immigrants and ethnoracial minorities in regions of the country that were most affected by the COVID-19 epidemic. While some of these explanations have been explored in the past, most researchers have only examined them in the context of the initial shock of the epidemic (except for Borjas and Cassidy [13]). Previous studies also usually assume that the effects of education or job characteristics on employment remain constant over time [9, 24–27].

Third, our analysis considers full-time employment as an alternative measure of the economic consequences of the COVID-19 epidemic. Previous studies have examined ethnoracial differences in employment [23], unemployment [7, 9] and labor force participation [22, 25]. Yet these measurements do not capture individuals who usually work full-time but experienced a reduction in work hours or were temporarily absent from work as a consequence of the shutdown [2, 28]. Our measure of full-time employment highlights the adverse effects of the epidemic that are not always captured by the rise in unemployment or the decline in labor force participation [29].

Finally, our analytical strategy accounts for seasonal or cyclical variations in employment of different demographic groups. The contraction in economic activity in the summer months following the onset of the epidemic in March, 2020 may be confounded with seasonal changes in the employment for some categories of workers [23, 29]. For example, labor demand in the agriculture, food processing and construction industries, which employ a larger percentage of immigrant workers, follows a clear seasonal pattern [30, 31].

### Differences in employment during the COVID-19 epidemic by immigrant status and race/ethnicity

Economic recessions have been shown to have an unequal effect across ethnoracial groups [32–34]. The employment of non-White workers is generally more sensitive to business cycles. During the Great Recession of 2008, for example, African American men had the highest unemployment rate of any group and were less likely to be rehired [32, 34]. Hispanics were at higher risk of reduced hours and unstable employment, especially those without postsecondary education [35, 36]. Asian Americans had the lowest underemployment rate during the Great Recession except in areas with a large construction sector [36, 37].

Recessions have also been shown to have a disproportionate impact on immigrant workers. Foreign-born workers are often the “first fired” during economic downturns but also the “first hired” when the economy recovers [10, 12]. During the Great Recession, foreign-born workers experienced a greater incidence of job loss [11, 38] and underemployment [36], particularly during the initial months.

It remains unclear whether the COVID-19 recession and its subsequent recovery affected the employment of different nativity and ethnoracial groups in the same way as previous recessions. On the one hand, the COVID-19 epidemic may be expected to have a greater impact on ethnoracial disparities because industries in the service sector that employ a disproportionate share of Hispanics and African Americans, such as leisure and hospitality, experienced the steepest declines in revenue and employment [15]. On the other hand, many firms in industries such as construction and transportation, which employ a large share of minority and immigrant workers, and were among the most affected during previous recessions, avoided large job losses during the COVID-19 lockdown because they were classified as essential by government agencies [17, 39].

Recent studies have found a disproportionate effect of the COVID-19 epidemic on the employment of immigrant workers consistent with the established hypothesis that immigrants are the “first fired” at the onset of an economic recession [10, 12]. Although immigrant men had higher employment rates than native men prior to the epidemic, they were more likely to become unemployed than native men by April 2020 [13]. Immigrant workers not only experienced higher levels of unemployment than the native-born, but also suffered a greater reduction in work hours during the first three months of the epidemic [40].

Comparatively more research has examined employment differences by race and ethnicity during the COVID-19 epidemic. These studies find substantial disparities across ethnoracial groups. Hispanic workers were the most adversely affected during the initial outbreak in the spring of 2020 [23]. They experienced the greatest increases in unemployment [7, 8, 19], and decreases in labor force participation [2]. Hispanics also had the highest incidence of financial difficulties and were more frequently unable to pay their bills in full [41]. Nevertheless, the unemployment level among Hispanic workers gradually declined during the summer of 2020 [7]. African-American workers experienced a smaller decline in employment compared to past recessions, yet they still had the second highest unemployment rate in April [19]. Couch et al. [7] find a widening unemployment gap between African American and White workers in May and June 2020, as the former group had more difficulties finding new jobs. Other studies document a recovery in employment levels after the reopening of the economy. By November 2020, the unemployment gap between minority workers and Whites seemed to have narrowed, but not closed [22].

Yet, little is known about how immigrant status is associated with employment across workers of different ethnoracial groups during the COVID-19 recession and subsequent recovery. Previous studies examining ethnoracial disparities in employment during this time period often do not disaggregate their analysis by immigrant status [7, 9, 23], while research on immigrants’ employment does not consider differences by race and ethnicity [13].

#### Potential explanations

Just as in the Great Recession, differences in human capital may contribute to ethnoracial disparities in employment during the epidemic. The COVID-induced recession appears to have had a more adverse effect on workers with less education, particularly those without a college degree [23]. Kim et al. [9] find that Asian Americans without a college degree experienced one of the greatest employment declines in the summer of 2020, while Asian American workers holding at least a Bachelor’s degree fared as well as Whites. Since fewer Black and Hispanic workers graduate from college [42, 43], education could partially account for their higher unemployment rates relative to Whites. Educational attainment is also closely related to workers’ occupation and may affect their likelihood of experiencing a job loss due to differences in the ability to telecommute [24, 44].

Second, differences in job characteristics might be another explanation for employment disparities by immigrant status and race/ethnicity during the epidemic. Low-skilled immigrant and minority workers are generally underrepresented in occupations that can be performed from home and thus were at greater risk of job loss during the COVID-19 shutdown [19, 44]. The ability to work remotely also varied by industry [8, 45]. For instance, individuals employed in food serving, beauty, fitness, and recreation services were less able to work remotely and consequently had a higher incidence of unemployment during the initial phase of the epidemic [24]. Borjas and Cassidy [13] found that the sharp declines in immigrants’ employment shortly after the COVID-19 outbreak can be partly attributed to the fact that their jobs are less likely to be performed from home. Results reported by Gezici and Ozay [19] suggest that Hispanic and Black men were no more likely to experience unemployment than White men in April 2020 once their ability to work from home was taken into account.

Finally, the geographic concentration of minority and immigrant workers in areas of the country that were hardest hit by the COVID-19 epidemic might also explain the disparities in employment [46, 47]. Hispanics and immigrant workers in particular are overrepresented in states such as California, New York, Florida and Texas that had high infection rates [20]. Likewise, Asian Americans are concentrated in the Pacific region where unemployment rates were higher [48]. States also varied in the timing, duration and intensity of government policies restricting social interaction and economic activities for preventing the spread of the virus. For example, California, New Jersey and Illinois were the first to implement mandatory stay-at-home orders in March 2020, while states in the southeast such as South Carolina, Missouri and Alabama did not issue such policies until later [21]. Reopening dates also varied largely by state and region.

In sum, although evidence suggests that both ethnoracial minority and immigrant workers were more likely to experience job loss during the COVID-19 recession, most studies have not examined the unequal impact on employment across immigrants of different ethnoracial groups as well as between immigrants and non-immigrants of the same race/ethnicity. In the analysis below, we examine the intersecting effect of immigrant status and race/ethnicity on employment differences during the COVID-19 epidemic through the end of 2021. This time frame allows us to consider the effect of the epidemic-induced recession and subsequent long-term recovery. We explore three potential explanations for the observed disparities across ethnoracial and immigrant status groups by testing whether they can be attributed to differences in educational attainment, job characteristics, and state of residence.

### Gender differences in employment during the COVID-19 epidemic

Past economic recessions have often resulted in a greater rise in unemployment among men than women [27, 49, 50]. By contrast, some studies suggest that female workers lost their jobs at higher rates during the COVID-19 epidemic [27, 51]. Landivar et al. [51] and Dias et al. [26] find a “fatherhood premium” as working mothers were more likely to be laid off after the outbreak of COVID-19 compared to fathers and childless workers [26, 51]. Other studies also find that mothers suffered greater declines in labor force participation and hours worked compared to fathers [27, 52, 53]. However, some of these disparities may be attributed to seasonal differences in employment by gender. Because the COVID-19 outbreak coincided with the summer months in which women’s employment often declines, estimates of gender disparities during the initial phase of the epidemic may capture customary seasonal fluctuations rather than the effect of the epidemic itself. Once seasonal fluctuations are taken into account, recent studies find no significant increase in gender disparities in full-time employment through the summer of 2020 [29, 54].

Previous studies have shown that women’s employment is affected by family characteristics including whether they are married, have children, and are living in households with others who are employed. Married women are less likely to be employed full-time than married men [55]. Women with children are also less likely to be full-time workers, especially when their children are young and when they have more than one child [55–58]. Women living in households with other employed members had lower rates of labor force participation in previous economic recessions [50, 59]. These factors may have also played a role during the COVID-19 epidemic. For example, Petts et al. [60] find that the increase in childcare and homeschooling responsibilities led to a greater risk of unemployment among women with young children. In the analysis below we therefore examine the effect of family characteristics on women’s employment during the COVID-19 epidemic.

## Data and methods

### Data

Our analysis uses harmonized data from the monthly panels of the CPS available through the University of Minnesota Population Center’s Integrated Public Use Microdata Series (IPUMS) [61]. The CPS has a 4-8-4 sampling design, where the selected households are interviewed for four consecutive months, rotate out of the survey for eight months, and then return for another four months before they permanently leave the sample. We select data from all months from 2019 to 2021. The monthly panels from 2020 and 2021 allow us to examine changes in individuals’ employment related to the COVID-19 epidemic during the entire two-year period, while the corresponding monthly panels from 2019 allow us to adjust for seasonal variation as described below.

Our analytical sample includes both men (N = 452,690) and women (N = 482,300) aged 25 to 55 who are not employed in the military in any wave of the survey. We exclude a small percentage of individuals whose gender changes across waves because we cannot ascertain whether it reflects an actual change in gender identity or a miscoding of the corresponding variable. Sample weights provided by the CPS are used throughout our analysis.

### Variables

We examine two measures of employment in our statistical models. First, we use the probability of unemployment as an outcome. Individuals are considered to be at risk of unemployment only if they are in the labor force. However, as noted earlier, unemployment is an imperfect measure to test the effects of COVID-19 because it does not capture individuals who exit the labor force, as well as individuals who experience a reduction in work hours or are temporarily not working [62]. We therefore include the probability of full-time employment as a second outcome in our statistical models. Full-time employment is defined as working 35 hours or more per week in all jobs combined [63]. Respondents who usually work full time but did not do so in the reference week are not considered full-time employees. This is a deliberate strategy that allows us to capture whether the COVID-19 epidemic led to a temporary pause or reduction in working hours for individuals who might still consider themselves employed full time [25]. Such disruptions may have meaningful consequences in individuals’ lives and have the potential of becoming permanent. Nevertheless, we conducted separate analyses in which individuals who usually work full time but were not working in the reference week were considered as employed full time. Results of these models are presented in S1 Table. We also tested models using the number of hours worked as an outcome, and present the results in S2 Table. Findings from these ancillary analyses were consistent with those presented below.

The primary predictors in our models are individuals’ ethnoracial identification and immigrant status. We distinguish four mutually-exclusive ethnoracial categories based on the information provided in the first interview: non-Hispanic White, non-Hispanic Black, non-Hispanic Asian, and Hispanic. Respondents classified as multiracial are assigned to the largest minority group with which they identify. We exclude a small number of cases of individuals classified as “other race and ethnicity.” We distinguish immigrant status based on the country of birth. Individuals are classified as immigrants if they were born outside the U.S. and outlying areas such as Puerto Rico. Children of U.S. parents born abroad are not considered immigrants. A small number of cases in which the country of birth varied across waves were excluded from our analysis.

Our models explore whether educational attainment, job characteristics and place of residence account for ethnoracial and immigrant status differences in employment in the months following the COVID-19 outbreak. As explained in the Methods section below, our fixed-effects models control for the effect of any individual characteristics that do not vary across waves. Educational attainment is therefore introduced by testing separate models for individuals with and without completed college defined as 16 years or more of formal schooling in the first interview. We also test models in which we control for the occupation, industry and state of residence by introducing an interaction term between each of these three variables and month of the year. Respondents’ occupation is defined using 76 occupational groups based on the Census Bureau’s 1990 occupational classification scheme. Respondents’ industry is grouped into fourteen major categories based on the Census Bureau’s 2012 industrial classification system.

### Methods

We use individual fixed-effects models to estimate differences in employment by race/ethnicity and immigrant status over all twelve months of 2020 and 2021. Linear probability models (LPM) are used to avoid problems when comparing coefficients across logistic regressions [64]. To examine differences across ethnoracial groups, we test separate models comparing Hispanic, Asian-American and African-American men and women to non-immigrant White men and women. We adjust for seasonality in employment across ethnoracial and nativity groups by estimating differences in employment each month relative to employment levels in the corresponding month of 2019. Seasonal fluctuations in employment may be especially strong for some categories of foreign-born workers such as those employed in agriculture and food processing [30, 31]. Women’s employment has also been found to decline during the summer months [29]. We explore differences in employment by immigrant status during the course of the COVID-19 epidemic in excess of employment disparities in 2019 by introducing a three-way interaction between individual’s immigrant status, the current month and year:

$$y_{ijk}=\beta_{0}+\beta_{1}^{\prime }{month}_{j}+\beta_{2}^{\prime }{year}_{k}+\beta_{3}^{\prime }{year}_{k}*{month}_{j}+\beta_{4}^{\prime }{immigrant}_{i}*{month}_{j}+\beta_{5}^{\prime }{immigrant}_{i}*{year}_{k}*{month}_{j}+{u_{i}+\varepsilon }_{ijk}$$

where *y_ijk_* is the measure of employment for individual *i* in month *j* for year *k*; **year_k_** is a vector of binary variables for each year using 2019 as the reference category; **month_j_** is a vector of binary variables for each month of the year; **immigrant_i_** is a vector of two binary variables indicating whether an individual *i* is a minority immigrant or a minority non-immigrant, leaving non-immigrant Whites as the reference group; *u_i_* are individual fixed effects; and *ε_ijk_* is an individual-month-year error term. Although the model includes a full three-way interaction between individuals’ immigrant status, the month of reference and the year, the term immigrant_i_*year_k_ is omitted by including an indicator for every month. The stand-alone term for **immigrant_i_** is also omitted from the fixed-effects models because individuals’ immigrant status does not vary over time. In this equation, the coefficients $\beta_{5}^{\prime }$ capture the excess in full-time employment or unemployment for immigrant and non-immigrant minority workers relative to native White workers adjusted for seasonality (i.e., compared to the same month in 2019). Standard errors are adjusted for clusters by individual.

After presenting the baseline estimates of ethnoracial disparities in employment, we examine differences by level of education using separate models for workers with and without a college degree. Finally, to test whether the concentration of minority and immigrant workers in different types of jobs or regions of the country explains the observed disparities in employment, we include ${industry}_{a}*{year}_{k}*{month}_{j},{occupation}_{b}*{year}_{k}*{month}_{j}$ and ${state}_{c}*{year}_{k}*{month}_{j}$ in our models. These three-way interaction terms fully capture any differences in employment for workers in each industry, occupation and state over time, and are therefore preferable to simply including indicators for industry, occupation and state as predictors. Such stand-alone predictors used in previous studies only capture time-invariant differences [9, 24–27]. For example, the interaction term between state of residence and month-year fully captures differences in employment over time across states that could have been brought about by state-level trends in infection rates and by government policies intended to curb the epidemic. Time-varying indicators such as infection rates or indexes of government restrictions are subject to measurement error and may miss important ways in which the epidemic affected employment levels over time.

Finally, to explore the effects of household characteristics on women’s employment during the COVID-19 epidemic we test separate models for: (a) women with and without a spouse or cohabiting partner; (b) women with and without children under 12; and (c) women living in households with and without any other full-time employed adults based on information from the previous wave of the survey. For symmetry, we also test the same models for men. Results from the corresponding models for men are presented in S2–S4 Tables to conserve space.

## Results

### Descriptive patterns

Table 1 shows a breakdown of 25–55 years-old workers of each ethnoracial group and immigrant status according to their industry and occupation, region of residence, and level of education. The descriptive statistics are calculated using the final outgoing rotation group in 2019, reflecting the pre-pandemic worker characteristics.

**Table 1:** Descriptive statistics of key variables by immigrant status and race/ethnicity among men aged 25–55 in the labor force (CPS 2019, wave 8).

	NB Whites	FB Blacks	NB Blacks	FB Hispanics	NB Hispanics	FB Asians	NB Asians
***Occupation***							
*Feasibility of remote working > 0*.*5*							
Management	14.5%	7.0%	8.1%	5.9%	8.9%	11.1%	10.5%
Computer and mathematics	3.7%	1.3%	3.1%	1.1%	2.4%	15.6%	8.7%
Office and administrative support	10.6%	9.6%	13.8%	7.2%	14.8%	7.6%	11.3%
Architecture and engineering	2.4%	1.1%	1.2%	0.9%	1.2%	4.6%	2.6%
Business and financial operations	6.1%	2.3%	4.6%	1.6%	3.7%	7.2%	9.4%
Educational instruction and library	7.8%	3.0%	5.4%	2.4%	5.1%	5.6%	4.9%
Arts, entertainment, sports and media	2.6%	1.0%	1.4%	1.0%	1.8%	1.8%	3.0%
Life, physical and social science	1.0%	0.9%	0.6%	0.4%	0.5%	2.3%	1.7%
Legal occupations	1.6%	0.4%	0.8%	0.4%	1.0%	0.5%	2.5%
** Subtotal**	**50.3%**	**26.5%**	**39.1%**	**21.0%**	**39.4%**	**56.3%**	**54.6%**
***Industry***							
Agriculture, forestry and fisheries	2.5%	0.4%	0.9%	7.5%	2.2%	0.4%	0.8%
Mining	0.6%	0.2%	0.3%	0.3%	0.6%	0.5%	0.1%
Construction	7.1%	4.1%	3.8%	19.7%	8.2%	1.7%	3.1%
Manufacturing	11.0%	7.6%	9.4%	10.7%	8.3%	11.7%	8.4%
Transportation and utilities	6.5%	12.4%	11.8%	7.1%	7.8%	7.6%	7.5%
Wholesale trade	2.4%	1.5%	1.7%	1.9%	2.8%	1.6%	2.1%
Retail trade	13.1%	13.2%	14.7%	17.4%	16.6%	15.4%	12.9%
Finance, insurance, real estate	8.0%	4.9%	5.9%	3.0%	7.2%	7.7%	7.4%
Business and repair services	7.9%	7.2%	8.1%	9.2%	8.2%	13.6%	12.8%
Personal services	2.3%	4.4%	3.2%	6.0%	3.0%	7.4%	3.3%
Entertainment and recreation	2.2%	0.7%	2.2%	1.3%	2.3%	1.4%	2.3%
Professional services	31.2%	42.0%	31.3%	14.1%	27.9%	28.3%	33.9%
Public administration	5.1%	1.4%	6.6%	1.7%	5.0%	2.6%	5.4%
***Region***							
Northeast	17.8%	35.6%	14.3%	13.2%	12.5%	18.6%	16.8%
Midwest	26.4%	12.2%	16.7%	10.0%	8.9%	13.6%	8.8%
South	36.0%	42.7%	59.6%	41.6%	37.0%	25.4%	19.9%
West	19.8%	9.5%	9.5%	35.3%	41.6%	42.4%	54.5%
***Education***							
No college degree	55.6%	64.1%	71.4%	85.2%	76.1%	36.2%	41.0%
College or more	44.4%	35.9%	28.6%	14.8%	23.9%	63.8%	59.0%
***Sex***							
Female	50.7%	54.3%	56.9%	51.1%	53.7%	54.2%	50.7%
Male	49.3%	45.7%	43.1%	48.9%	46.3%	45.8%	49.3%

As discussed previously, all foreign-born minority groups were more likely than their native-born counterparts to be employed in the retail trade and personal services industries, which experienced the largest increases in unemployment compared to 2019. At the same time, Hispanic workers were more likely to be employed in construction, an industry less affected by the economic downturn. Black and Hispanic workers, especially the foreign-born, were also less frequently employed in occupations for which working from home is more feasible. The feasibility of working remotely is assessed using an index created by Dingel and Neiman [45] based on the O*NET classification system. While 45.1% of White men worked in occupations with high feasibility of remote working, only 31.9% of African Americans and 23.2% of Hispanics worked in such occupations. The proportion of workers who can work remotely was largest among Asian Americans primarily due to their overrepresentation in occupations related to computer and mathematics as well as architecture and engineering.

Table 1 also shows differences in the regional distribution of the working-age population by race/ethnicity and immigrant status. African Americans and foreign-born Hispanics were more concentrated in the Southern and Western census regions, while native-born Hispanics and Asian Americans were disproportionately concentrated in the Western region. Finally, we also observe large disparities in educational attainment consistent with findings from previous studies [65]. Hispanics and native-born Blacks in general had significantly lower levels of college completion. These preexistent ethnoracial differences in socioeconomic characteristics could potentially affect the employment prospects of minority and immigrant workers following the onset of the COVID-19 epidemic.

**Fig 1:**
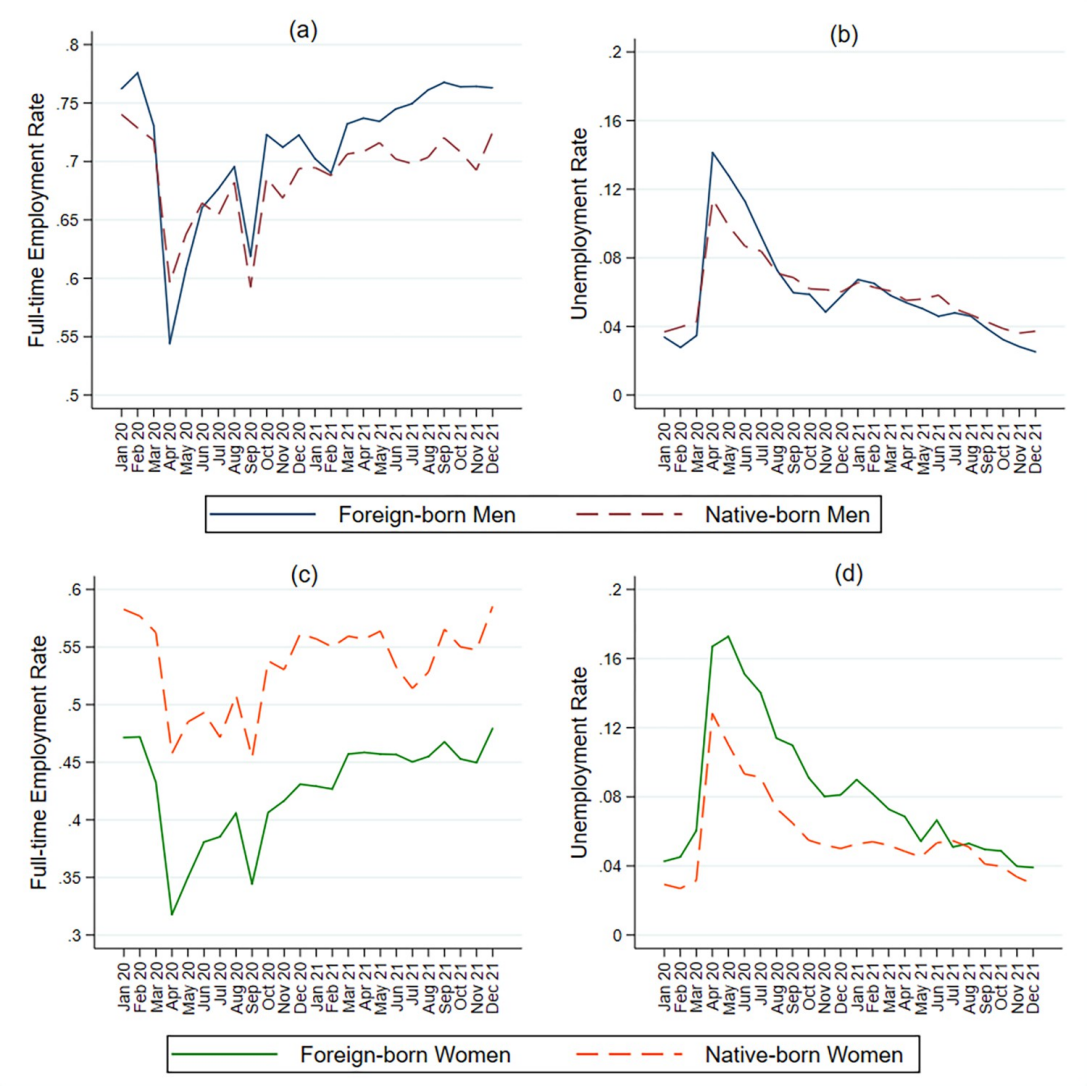
Employment levels of foreign-born and native-born men and women, January 2020-December 2021.

**Fig 2:**
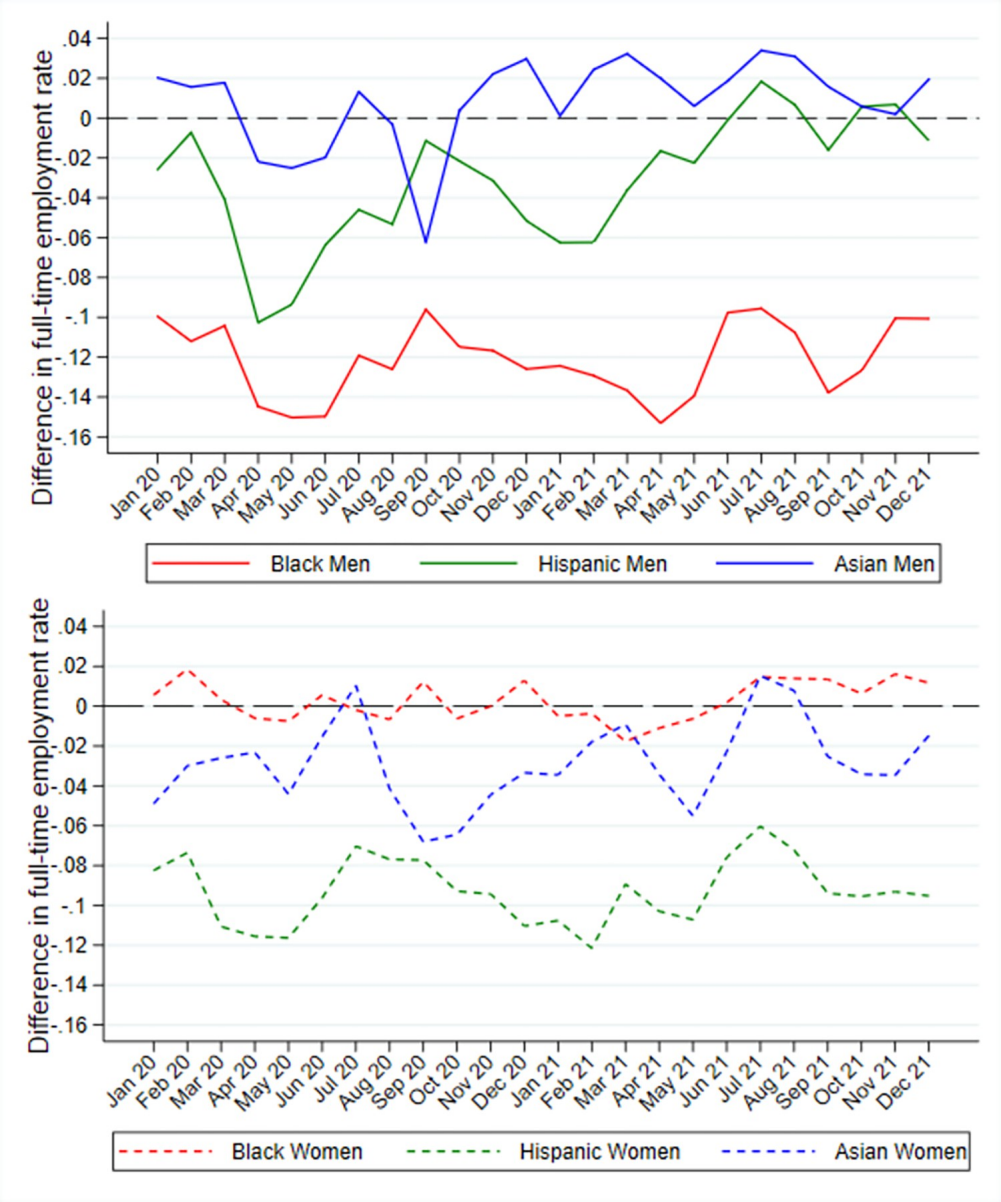
Difference in full-time employment rate for minority men and women relative to Whites, January 2020-December 2021.

Fig 1 shows the full-time employment and unemployment rates for foreign-born and native-born men from January 2020 to December 2021, while Fig 2 shows the difference in full-time employment for minority men relative to White men. Immigrant men experienced greater decreases in employment than native-born men starting in April 2020. The gap in full-time employment between White men and non-White men also increased dramatically in the early months of the epidemic. Hispanic men experienced the largest decrease in full-time employment, although full-time employment was lowest among African Americans. The full-time employment rate of Asian-American men fell below that of Whites from April to June 2020.

However, during the late-summer and early-fall of 2020, men of all ethnoracial minority groups experienced a relative recovery in employment. By the end of 2020, differences in employment levels between immigrant men and native-born men as well as those between Asian men and White men had largely reverted to their pre-pandemic levels. By contrast, Black men and Hispanic men continued to experience a wider gap in full-time employment relative to Whites in December 2020. Despite some fluctuations, ethnoracial disparities resemble the pre-pandemic pattern throughout most of 2021. Foreign-born men have consistently higher full-time employment rates in all months of 2021. The surge in infection rates due to the introduction of the Delta variant of the COVID-19 virus in the late summer of 2021 does not appear to significantly affect differences in employment by immigrant status, but might have led to larger differences in full-time employment between ethnoracial minority men and White men.

Women’s employment during the COVID-19 epidemic shows a different pattern from men. Fig 1(C) and 1(D) indicate that foreign-born women experienced consistently lower full-time employment levels and higher unemployment levels than native-born women throughout 2020 and 2021. The decline in full-time employment for foreign-born women was larger than for native-born women during the initial months of the epidemic. The gap in employment between foreign-born and native-born women appears to decrease to its pre-pandemic level by early 2021. Fig 2 indicates that Hispanic women had the lowest full-time employment rate among non-White women compared to their White counterparts. The largest decline in full-time employment was experienced by Asian women between July and September of 2020. By the end of 2020, the gap in full-time employment between Black and White women and between Asian and White women had reverted to their pre-pandemic levels. However, Hispanic women still experienced a larger gap in full-time employment relative to White women.

### Baseline estimates

**Fig 3:**
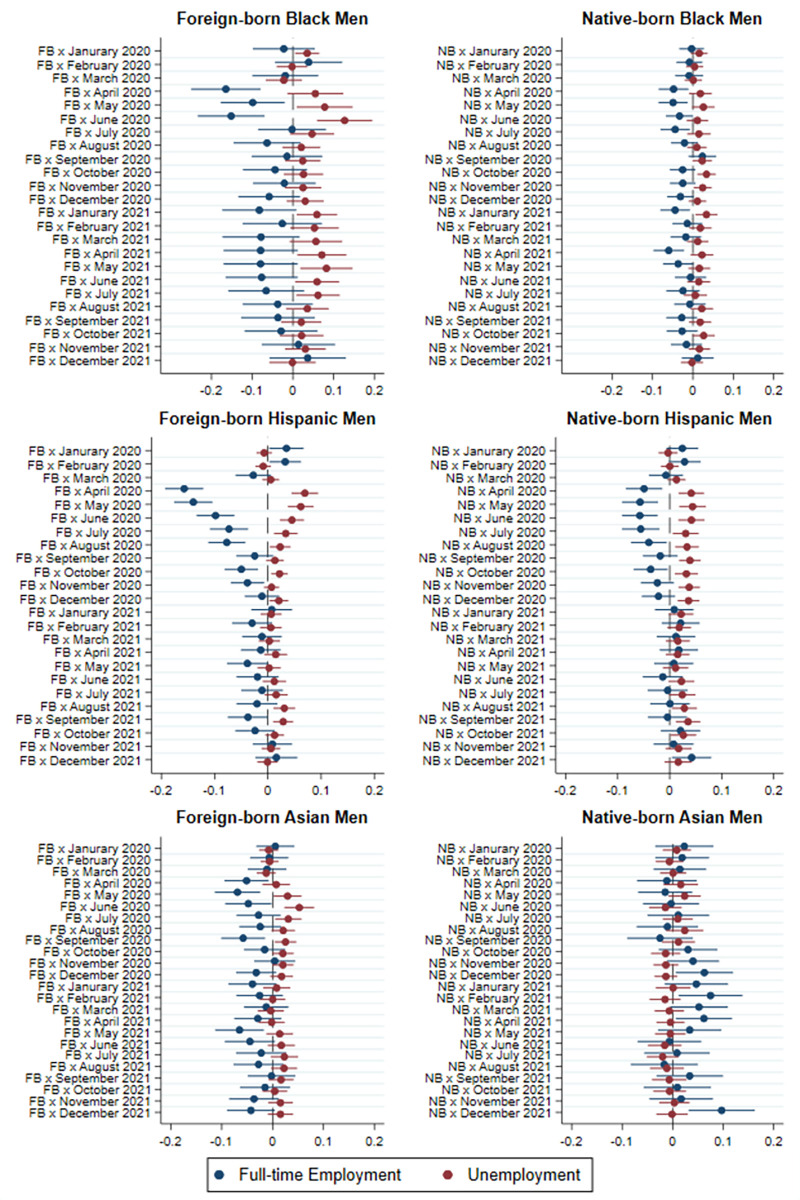
Difference in probability of full-time employment and unemployment between non-White and native-born White men by immigrant status and race/ethnicity, 2020–2021 relative to 2019.

Fig 3 presents the results of baseline models comparing the probability of full-time employment and unemployment of foreign-born and native-born men of each race and ethnicity relative to native-born Whites. The predicted differences in probabilities are based on the models including the three-way interaction terms between individuals’ immigrant status, the current month and year. As discussed in the methods section these models adjust for seasonality in individuals’ employment. Separate graphs are presented for foreign- and native-born men of each ethnoracial group because they are each being compared against the reference category of native-born Whites.

The results in the left panel of Fig 3 indicate that immigrant men experienced a significantly greater decrease in full-time employment and an increase in unemployment compared to native White men *only* during the early months of the epidemic in 2020. The difference between immigrant men in each ethnoracial category and native Whites is larger in the models for full-time employment than unemployment. However, the employment gap between immigrant men and native Whites declined quickly after June 2020, and Hispanic immigrants were the only group among the foreign born that still experienced significantly lower full-time employment rates and higher unemployment compared to native Whites by the end of the summer of 2020.

The right panels of Fig 3 indicate that non-immigrant minority men experienced a lower excess risk of non-full-time employment and unemployment than immigrant men in the months following the onset of the epidemic. Just as their immigrant co-ethnic counterparts, Hispanic non-immigrant men had the greatest and longest decline in employment of any non-immigrant ethnoracial group. However, they had significantly lower excess risk of non-full-time employment compared to Hispanic immigrant men.

**Fig 4:**
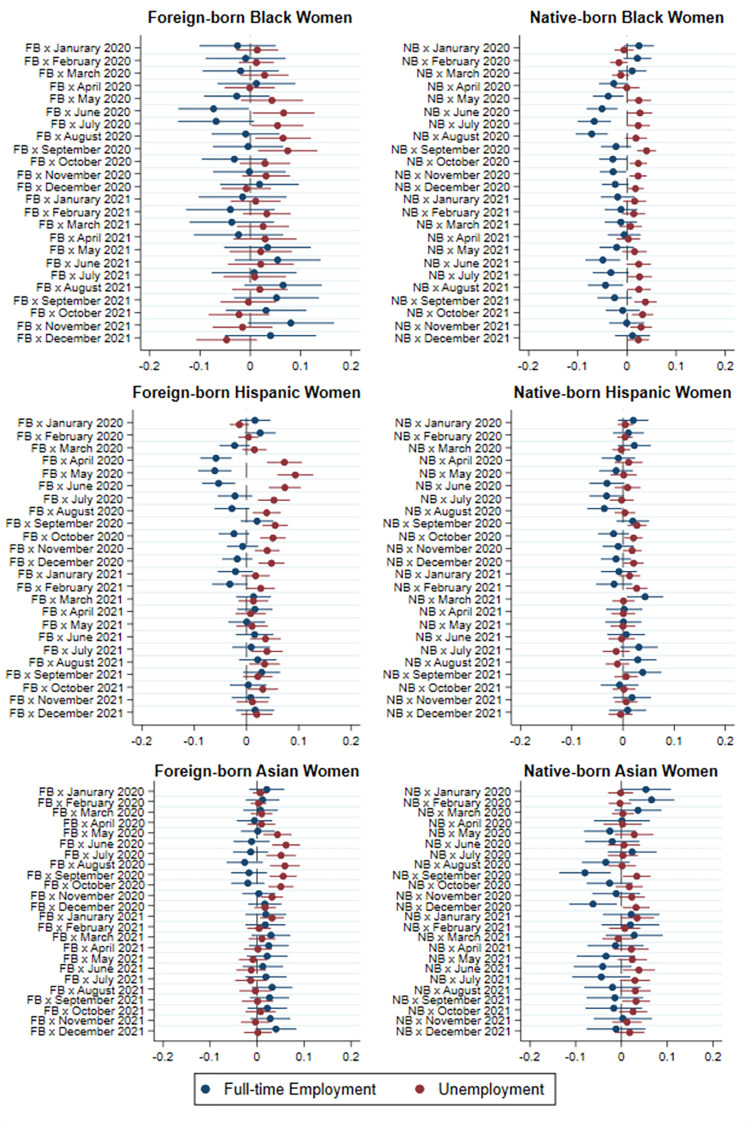
Difference in probability of full-time employment and unemployment between non-White and native-born White women by immigrant status and race/ethnicity, 2020–2021 relative to 2019.

Fig 4 presents the results of the baseline model for women in graphical form. Once again, only the difference in the probability of full-time employment and unemployment relative to native-born White women is presented in each graph. The results in the left panel show that all foreign-born women experienced higher unemployment levels compared to native-born Whites after the initial outbreak of COVID-19. The differences in unemployment only lasted through the summer months of 2020 for foreign-born Black and Asian women, but persisted until February 2021 for foreign-born Hispanic women. Foreign-born Black and Hispanic women also experienced lower full-time employment levels than native-born Whites during the initial months of the COVID-19 outbreak. The right-hand panel of Fig 4 indicates that native-born non-White women had a much lower risk of non-full-time employment and unemployment than women who are foreign born. While native-born Black women did have a lower rate of full-time employment and a higher rate of unemployment from May 2020 until the end of the year, there was no significant decline in employment among native-born Hispanic and Asian women compared to Whites.

Our comparison of immigrant workers with their non-immigrant counterparts of the same ethnoracial group in Figs 3 and 4 allows us to isolate the effect of immigrant status from that of race and ethnicity. Once we control for individual’s ethnoracial identification, we find that immigrant status only increased the risk of non-full-time employment and unemployment in the early months of the epidemic, namely from April to June 2020. After those initial months, the lower employment rates among immigrant workers can be largely attributed to their race and ethnicity. In models presented in S1 Fig in which all immigrants are grouped together without distinguishing their race and ethnicity, we find stronger and more long-lasting effects of the epidemic on immigrant-native disparities in employment. In these models, immigrants appear to experience lower employment levels than natives through the fall of 2020. Thus, failing to disaggregate immigrants by race and ethnicity leads to an overestimation of the effects of the COVID-19 epidemic on full-time employment and unemployment of foreign-born individuals.

### Women’s employment and household characteristics

Since family dynamics have been shown to play an important role in women’s employment, we investigate the extent to which their employment status changed over the course of the COVID-19 epidemic according to whether they had a marital or cohabiting partner, children living in the household and other household members who are employed. Tables 2–4 present the coefficients for the three-way interaction between individuals’ immigrant status and the current month and year for women according to these three household characteristics. The coefficients reflect the change in the probability of full-time employment for immigrant and non-immigrant women by race/ethnicity, respectively compared to native-born White women.

**Table 2:** Difference in the probability of women’s full-time employment relative to native-born Whites by immigrant status, race/ethnicity and marriage/cohabitation in 2020 relative to 2019.

	Married/cohabiting			Not married/cohabiting		
	Black	Hispanic	Asian	Black	Hispanic	Asian
Foreign-born*Month 2020						
Foreign-born*January 2020	-0.027	0.012	0.001	-0.019	0.029	0.129**
Foreign-born*February 2020	-0.048	0.026	0.003	0.047	0.027	0.073
Foreign-born*March 2020	-0.051	-0.021	-0.007	0.014	-0.022	0.089
Foreign-born*April 2020	-0.090	-0.038*	-0.024	0.147*	-0.108**	0.043
Foreign-born*May 2020	-0.069	-0.033	0.011	0.042	-0.114**	-0.060
Foreign-born*June 2020	-0.061	-0.028	0.006	-0.083	-0.109**	-0.112*
Foreign-born*July 2020	-0.117*	-0.012	-0.005	0.012	-0.044	-0.083
Foreign-born*August 2020	-0.030	-0.019	-0.033	0.051	-0.049	-0.022
Foreign-born*September 2020	-0.039	0.017	-0.017	0.055	0.030	-0.037
Foreign-born*October 2020	-0.034	-0.022	-0.032	-0.013	-0.017	0.029
Foreign-born*November 2020	-0.024	-0.015	0.005	0.056	0.010	-0.007
Foreign-born*December 2020	0.023	-0.020	0.015	0.042	-0.020	0.016
Native-born*Month 2020						
Native-born*January 2020	0.029	0.001	0.077*	0.019	0.051*	0.023
Native-born*February 2020	-0.001	-0.001	0.048	0.024	0.023	0.086*
Native-born*March 2020	-0.005	0.011	0.034	0.008	0.029	0.049
Native-born*April 2020	-0.041	-0.016	-0.005	0.003	0.017	0.019
Native-born*May 2020	-0.038	-0.014	-0.001	-0.018	-0.001	-0.049
Native-born*June 2020	-0.046	-0.030	-0.006	-0.045	-0.026	-0.041
Native-born*July 2020	-0.060*	-0.026	0.025	-0.050*	-0.026	0.036
Native-born*August 2020	-0.081**	-0.038	-0.044	-0.043	-0.022	0.002
Native-born*September 2020	-0.040	0.024	-0.080*	-0.000	0.014	-0.073
Native-born*October 2020	-0.012	-0.014	-0.037	-0.014	-0.016	-0.005
Native-born*November 2020	-0.026	-0.026	-0.010	0.003	0.030	-0.003
Native-born*December 2020	-0.020	-0.017	-0.050	-0.007	0.002	-0.075
Constant	0.570***	0.536***	0.555***	0.577***	0.579***	0.592***
Observations	368141	413277	377332	176411	165226	139578
Adjusted R-squared	0.639	0.647	0.646	0.616	0.610	0.620

Notes

*p < .05

**p < .01

***p < .001

**Table 3:** Difference in the probability of women’s full-time employment relative to native-born Whites by immigrant status, race/ethnicity and parenthood in 2020 relative to 2019.

	With children			Without children		
	Black	Hispanic	Asian	Black	Hispanic	Asian
Foreign-born*Month 2020						
Foreign-born*January 2020	-0.025	0.005	0.025	-0.015	0.026	0.038
Foreign-born*February 2020	-0.036	0.005	0.026	0.039	0.021	0.019
Foreign-born*March 2020	-0.044	-0.027	-0.022	0.018	-0.020	0.002
Foreign-born*April 2020	-0.049	-0.056*	0.001	-0.069	-0.134***	-0.099*
Foreign-born*May 2020	-0.068	-0.042	0.035	0.002	-0.113***	-0.057
Foreign-born*June 2020	-0.092	-0.036	0.039	-0.141*	-0.072*	-0.046
Foreign-born*July 2020	-0.101	0.001	-0.010	-0.105	-0.062*	-0.048
Foreign-born*August 2020	0.011	-0.004	-0.010	-0.008	-0.054	-0.121**
Foreign-born*September 2020	0.029	0.020	-0.019	-0.075	0.029	-0.028
Foreign-born*October 2020	-0.030	-0.033	0.011	-0.013	-0.026	-0.080*
Foreign-born*November 2020	-0.002	-0.024	0.010	-0.143*	-0.004	-0.014
Foreign-born*December 2020	-0.020	-0.021	0.024	-0.094	-0.044	0.028
Native-born*Month 2020						
Native-born*January 2020	0.021	0.048*	0.043	0.026	-0.038	0.112
Native-born*February 2020	0.013	0.013	0.087*	0.009	0.010	-0.044
Native-born*March 2020	0.034	0.027	0.007	-0.007	0.013	-0.004
Native-born*April 2020	-0.014	-0.030	-0.037	-0.069*	-0.011	0.034
Native-born*May 2020	-0.075*	-0.034	-0.012	-0.034	0.004	-0.047
Native-born*June 2020	-0.073*	-0.052	-0.012	-0.052	-0.042	-0.013
Native-born*July 2020	-0.071*	-0.059*	0.070	-0.074*	-0.044	-0.105
Native-born*August 2020	-0.076*	-0.042	0.004	-0.073*	-0.079*	-0.103
Native-born*September 2020	-0.070*	0.018	-0.066	0.003	-0.006	-0.093
Native-born*October 2020	-0.025	-0.002	-0.054	-0.064*	-0.103**	-0.062
Native-born*November 2020	-0.015	-0.012	-0.014	-0.088**	-0.082*	-0.105
Native-born*December 2020	-0.051	-0.027	-0.048	-0.034	-0.076*	-0.055
Constant	0.487***	0.449***	0.466***	0.596***	0.567***	0.578***
Observations	187388	209177	180884	122584	133894	114734
Adjusted R-squared	0.641	0.651	0.655	0.636	0.634	0.642

Notes

*p < .05

**p < .01

***p < .001

**Table 4:** Difference in probability of women’s full-time employment relative to native-born Whites by immigrant status, race/ethnicity and employment status of other household members in 2020 relative to 2019.

	Other hsld. members employed			No hsld. members employed		
	Black	Hispanic	Asian	Black	Hispanic	Asian
Foreign-born*Month 2020						
Foreign-born*January 2020	-0.013	0.045*	0.007	-0.022	0.04	0.013
Foreign-born*February 2020	-0.025	0.037	0.004	-0.067	-0.018	0.011
Foreign-born*March 2020	-0.08	-0.013	0.011	0.031	-0.015	0.061
Foreign-born*April 2020	-0.065	-0.038	0.034	0.048	-0.112**	-0.037
Foreign-born*May 2020	0.003	-0.012	0.007	-0.084	-0.114**	0.031
Foreign-born*June 2020	-0.039	-0.045	-0.002	-0.142*	-0.103**	-0.032
Foreign-born*July 2020	-0.072	-0.004	-0.01	-0.116*	-0.046	0.011
Foreign-born*August 2020	0.035	-0.028	-0.031	0.003	0.004	0.049
Foreign-born*September 2020	-0.005	0.013	-0.015	0.026	0.041	0.01
Foreign-born*October 2020	-0.035	-0.025	-0.018	-0.032	-0.024	-0.06
Foreign-born*November 2020	0.006	-0.027	0.007	-0.045	0.041	0.007
Foreign-born*December 2020	-0.04	-0.018	0.012	0.025	-0.029	-0.011
Native-born*Month 2020						
Native-born*January 2020	0.017	0.003	0.047	0.066**	0.061*	0.048
Native-born*February 2020	-0.004	0	0.108*	0.027	0.047	0.035
Native-born*March 2020	0.035	0.042	0.039	0.007	0.02	0.085
Native-born*April 2020	-0.021	0.013	0.012	-0.061*	0.003	0.032
Native-born*May 2020	-0.036	-0.002	-0.038	-0.045	0.011	0.087
Native-born*June 2020	-0.054	-0.013	-0.032	-0.060*	-0.083*	0.015
Native-born*July 2020	-0.086**	-0.033	-0.021	-0.042	-0.058	0.136*
Native-born*August 2020	-0.056	-0.016	-0.051	-0.076**	-0.039	0.007
Native-born*September 2020	-0.04	0.02	-0.081	-0.013	0.014	-0.077
Native-born*October 2020	-0.017	0.005	-0.041	-0.033	-0.058	0.032
Native-born*November 2020	-0.017	-0.002	-0.033	-0.027	0.013	-0.103
Native-born*December 2020	-0.029	-0.01	-0.071	-0.04	-0.033	-0.121*
Constant	0.614***	0.580***	0.601***	0.523***	0.513***	0.533***
Observations	236340	265645	240342	143225	137277	122054
Adjusted R-squared	0.651	0.658	0.658	0.645	0.645	0.649

Notes

*p < .05

**p < .01

***p < .001

Overall, the results are consistent with our earlier findings for men. Even after accounting for household characteristics, foreign-born women experienced significantly lower employment levels than their native-born peers of the same race/ethnicity, but mostly during the early months of the COVID-19 outbreak.

The results also indicate that the employment gap between Black and Hispanic women relative to Whites is actually *lower* for those with a partner, young children, or another full-time employed household member. This finding is consistent with previous work on gender disparities in employment for all women combined during the COVID-19 epidemic after controlling for seasonality [29]. Differences in family characteristics explain a large part of the observed disparities among Hispanic women by immigrant status, but they do not appear to explain the disparities for Black and Asian immigrant women relative to their non-immigrant counterparts. Hispanic immigrant women who have resident partners, young children, or live with another full-time employed household member experienced almost no greater employment disadvantages during the COVID-19 epidemic compared to both native-born Hispanic women and native-born White women.

### Differences by education, industry/occupation and region

**Fig 5:**
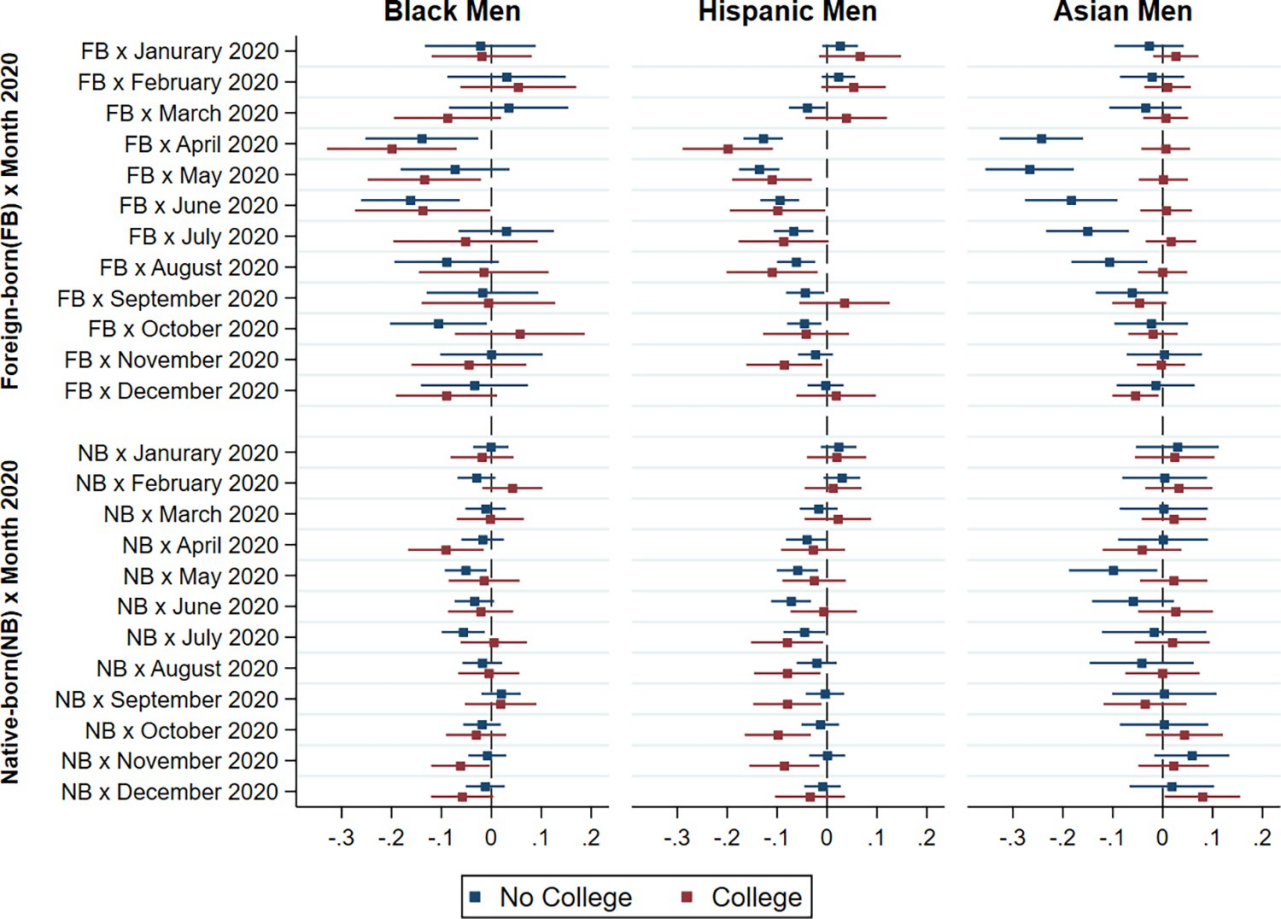
Difference in probability of full-time employment between non-White and native-born White men by immigrant status, race/ethnicity and education in 2020 relative to 2019.

**Fig 6:**
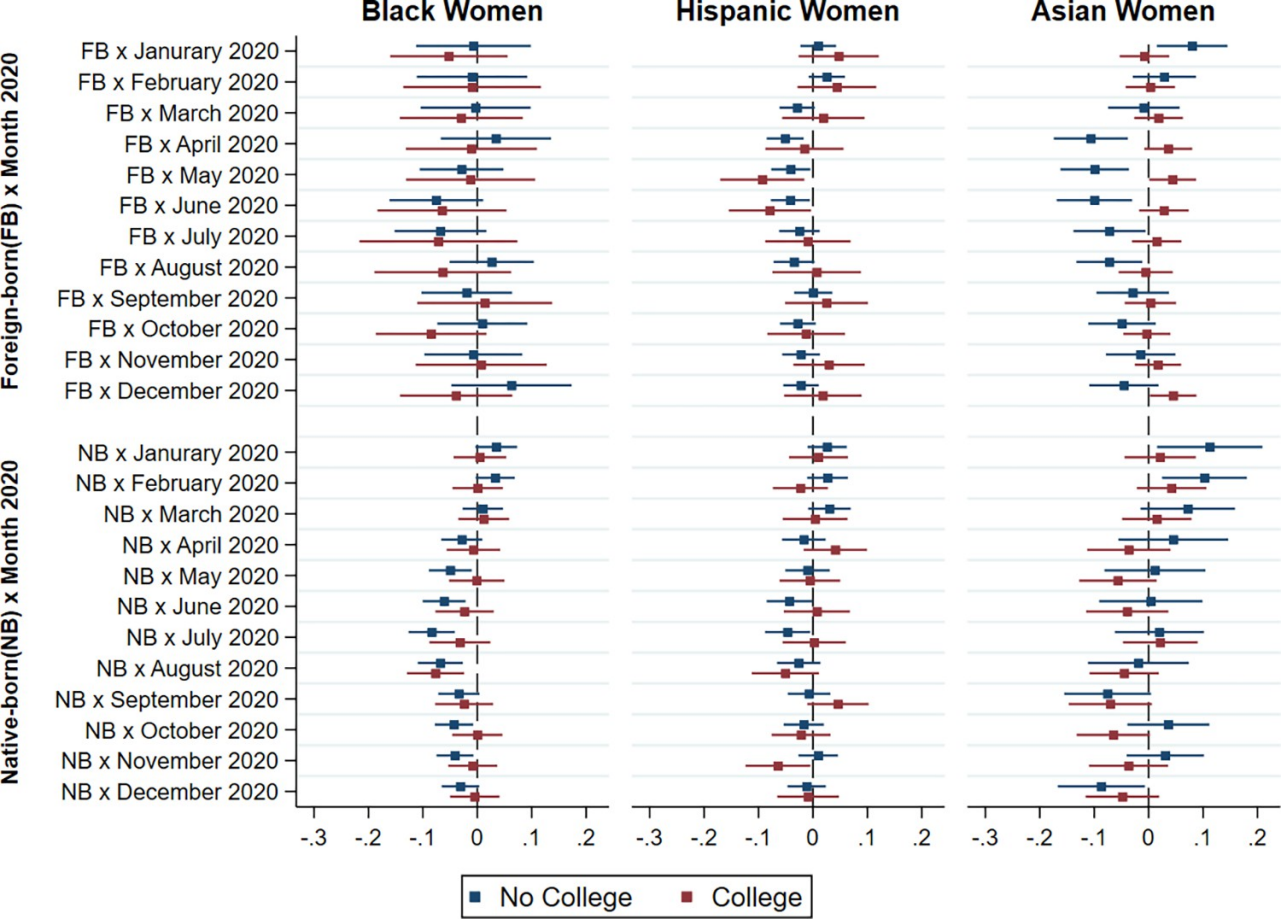
Difference in probability of full-time employment between non-White and native-born White women by immigrant status, race/ethnicity and education in 2020 relative to 2019.

To examine whether the observed disparities in the risk of not being employed full-time during the COVID-19 epidemic can be attributed to differences in educational attainment, we tested separate models for men with and without college degree in each ethnoracial group relative to native-born Whites. The results of these regression models are shown in Figs 5 and 6 in graphical form. For the sake of parsimony, we limit this part of our analysis to 2020, the year for which we find greater differences in full-time employment and unemployment by race/ethnicity and immigrant status. We find that differences in education account for a large part of the disparities in full-time employment following the COVID-19 outbreak. Specifically, education appears to account for the larger risk of non-full-time employment among Asian American immigrant men and women.

However, educational attainment does not explain the greater effect of the epidemic on the employment of Hispanic and African-American men and women. While previous studies using datasets other than the CPS, such as the Panel Study of Income Dynamics (PSID) [24] and the Survey of Household Economics and Decision-making (SHED) [41] found that non-White workers and the less-educated are more economically vulnerable, which are consistent with our findings, our analysis on employment disparities interacting immigrant status and race/ethnicity shows that Hispanic and African American immigrant men with a college degree actually experienced an even larger gap in full-time employment than their less-educated peers of the same ethnoracial group, when both being compared to native Whites with the same level of education. Therefore, the results presented in Figs 5 and 6 again suggest that the epidemic had its largest effect on the employment of immigrant minority men rather than their non-immigrant counterparts, even after controlling for their educational attainment.

**Fig 7:**
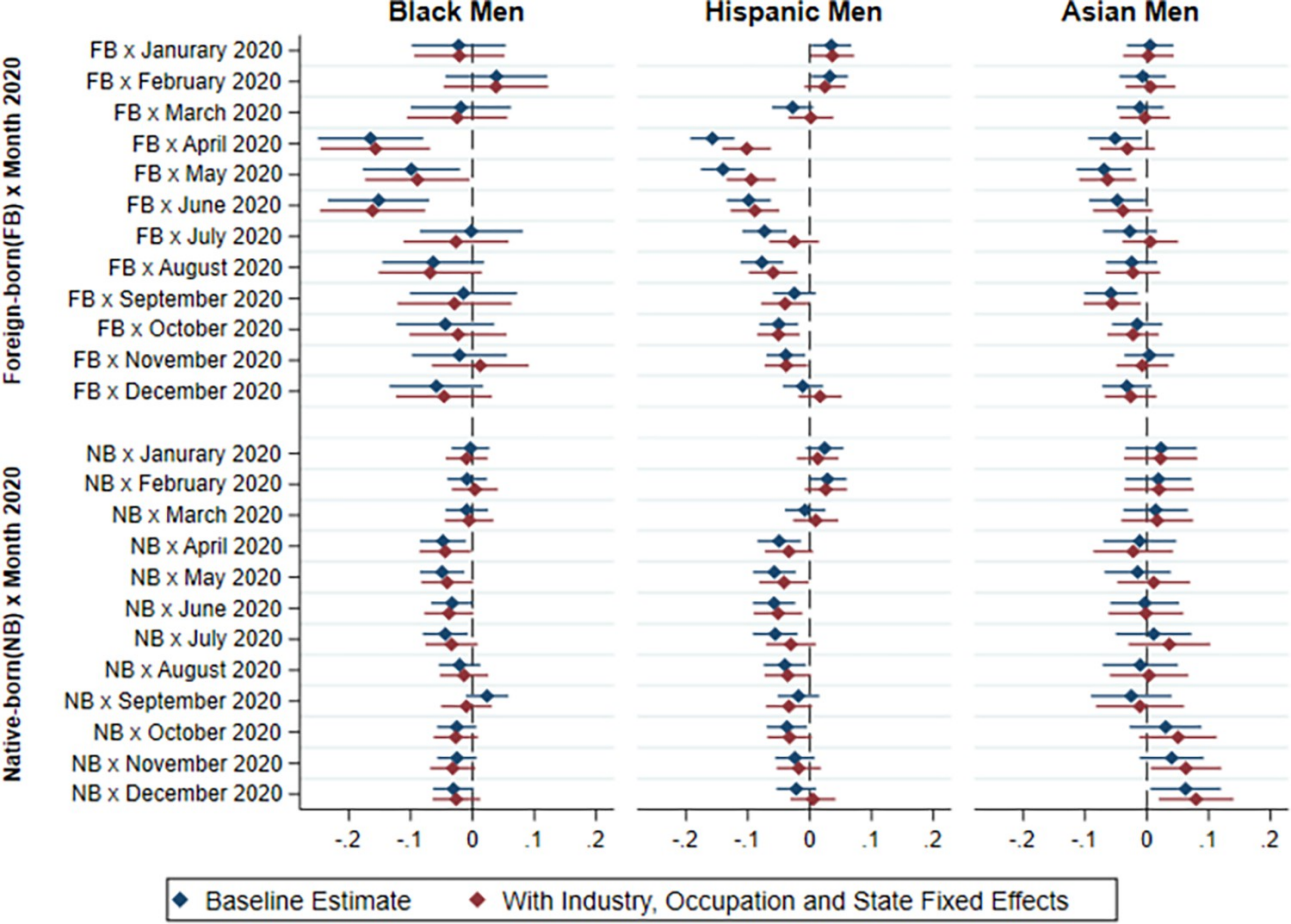
Difference in probability of full-time employment between non-White and native-born White men by immigrant status and race/ethnicity in 2020 relative to 2019 (with industry, occupation and state fixed effects).

**Fig 8:**
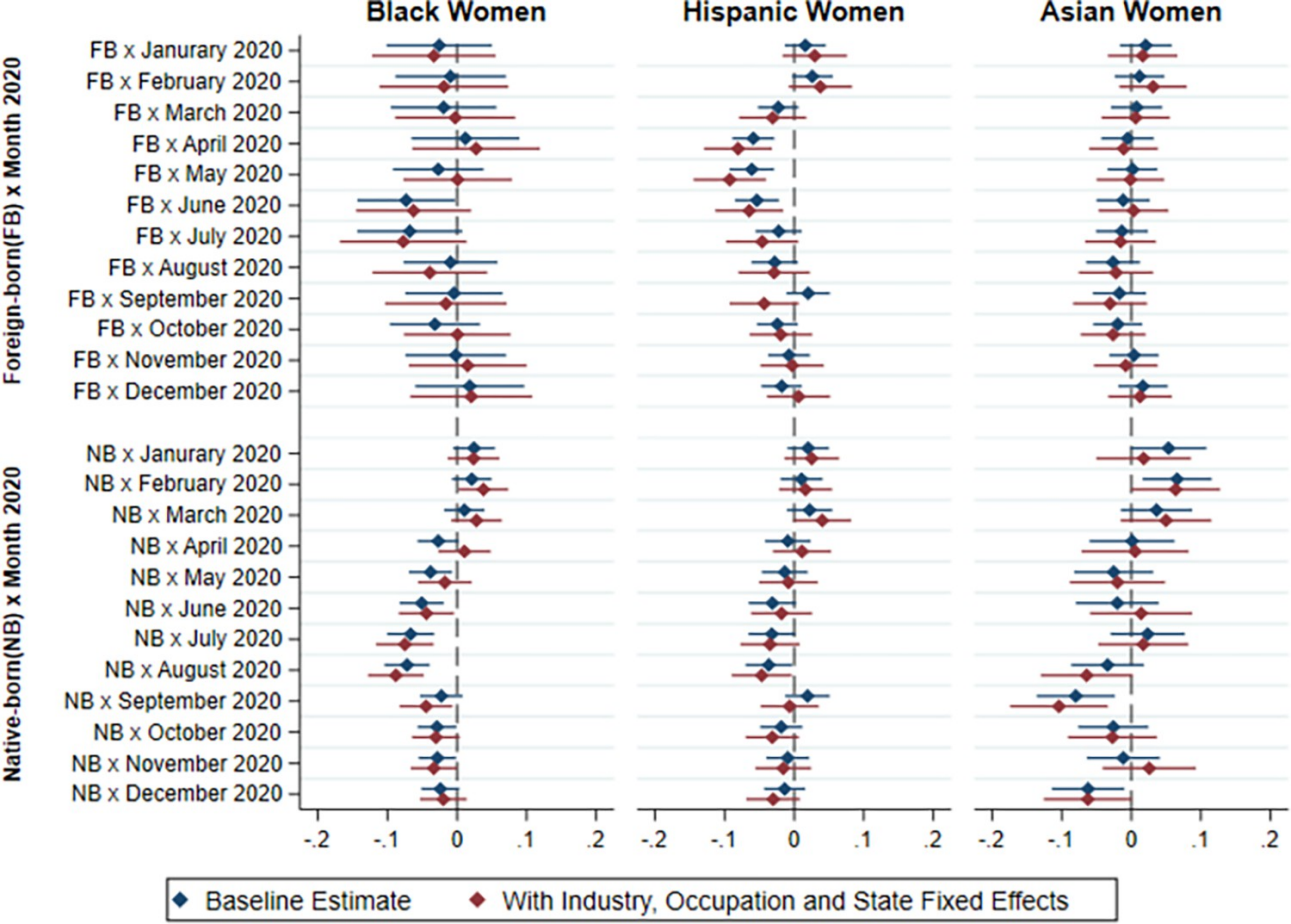
Difference in probability of full-time employment between non-White and native-born White women by immigrant status and race/ethnicity in 2020 relative to 2019 (with industry, occupation and state fixed effects).

Next, we examine the extent to which differences in employment by race/ethnicity and immigrant status during the COVID-19 epidemic are explained by the greater concentration of minority and immigrant workers in industries, occupations and regions of the country that were harder hit. As explained in the methodological section, we include interaction terms between industry, occupation and state of residence respectively, with the month and year. Figs 7 and 8 show the results of our models including these interaction terms for African Americans, Hispanics, and Asian Americans respectively, using non-immigrant Whites as the reference category. The results show that the ethnoracial and immigrant status differences in the baseline models persist, although the magnitude of the differences is somewhat attenuated after including the industry, occupation and state fixed-effects interactions with month and year. Our findings therefore suggest that the higher risk of not being employed full time among minority and immigrant men and women cannot be fully explained by the industry and occupation in which they are employed, or their concentration in particular states that were harder hit by the epidemic.

## Conclusions

In this paper, we examined ethnoracial and immigrant status differences in employment during the COVID-19-induced recession and recovery. Our analysis considered the impact of the epidemic from January 2020 to December 2021. We found that the ethnoracial disparities in employment during the epidemic were less long-lasting than in past recessions, with the notable exception of the gap between Hispanic and White men, which persisted throughout 2020. The effect of the epidemic on African Americans was smaller and shorter-lived compared to other recessions. By the fall of 2020, disparities in both unemployment and full-time employment for Black and Asian men relative to White men had largely reverted to their pre-epidemic levels. The same is true for women of all ethnoracial minority groups.

Our analysis highlights the importance of considering the nexus between race/ethnicity and immigrant status in employment outcomes. The employment disparities between immigrant and non-immigrant workers during the COVID-19 epidemic found in earlier studies appears to be driven in large part by the overrepresentation of Hispanics among the foreign-born. Once we disaggregate immigrants according to their race/ethnicity, we find the increased disparity in employment by immigration status to be remarkably short lived. After adjusting for seasonality, foreign-born individuals only experienced higher rates of unemployment and lower rates of full-time employment in the first months of the epidemic. Failing to disaggregate immigrants by race and ethnicity therefore leads to an overestimation of the effects of the COVID-19 epidemic on the employment of foreign-born individuals.

Our study examined full-time employment as an alternative measure of the economic consequences of the COVID-19 epidemic. Previous studies mostly rely on the unemployment rate as a key indicator. However, the unemployment rate does not capture the extent to which individuals withdraw from the labor force, reduce the number of hours they work or declare themselves to be employed even though they are not currently working. Interestingly, the disparity by immigrant status was larger when measured using full-time employment than unemployment, suggesting that there was a substantial number of immigrant men and women who became underemployed or dropped out from the labor force entirely. By contrast, the disparity between Hispanic and White workers was longer-lasting when measured using the unemployment rate rather than the rate of full-time employment. This suggests that compared to native White men, Hispanic men experienced a comparatively greater probability of becoming unemployed than exiting the labor force or having their hours reduced.

We also considered several alternative explanations for the observed ethnoracial and immigrant status disparities in employment. First, we found that differences in human capital, as measured by the level of education, only partly explain the observed disparities. Asian-American immigrants with a college degree were not at higher risk of losing their full-time employment compared to native Whites. However, African-American men and Hispanic men and women continued to experience a higher risk of non-full-time employment regardless of immigrant status even when we accounted for their educational attainment.

Second, we also considered whether the observed disparities in full-time employment could be explained by the concentration of minority and immigrant workers in occupations, industries and regions of the country that were most affected by the COVID-19 epidemic. Our results indicated that *none* of these factors alone or in combination fully explain the observed disparities in employment. We also investigated how women’s employment in particular might be influenced by family characteristics. We found that having a partner, young children, or another full-time employed worker in the household partly explain the immigrant-native gap in employment among Hispanic women, but not among Black and Asian women.

Why then did the COVID-19 epidemic have a greater effect on the employment of minority and immigrant workers, particularly in the initial months of the epidemic? It is possible that the jobs of minority and immigrant workers are more precarious in ways that are not fully captured by our indicators of industry and occupation. It is also possible that states are geographical units that are too large to properly capture differences in the effect of the epidemic by place of residence. Another possibility is that minority workers benefited less from government policies intended to alleviate the effect of the epidemic. Chetty et al. [66] found that multiple stabilization policies including state-ordered reopening, stimulus payments and the Paycheck Protection Program loans only had a small impact on employment recovery. Because non-citizen and undocumented workers are often ineligible for these benefits, they may have had no alternative but to continue working full time even at the risk of exposure to COVID-19 infection. Finally, the greater effect of the epidemic-induced recession on minority workers might reflect discriminatory practices [67, 68]. Future research should aim to refine the measures of job characteristics, further isolate the possible effect of discrimination and more thoroughly examine how stabilization policies might have affected immigrant and ethnoracial minority workers differently.

## Supporting information

S1 Fig Difference in probability of full-time employment and unemployment between immigrants and non-immigrants, 2020–2021 relative to 2019. (TIF)

S1 Table Robustness check: Counting full-time employed individuals not working full-time at the time of interview as full-time workers. Notes: *p < .05, **p < .01, ***p < .001.
(PDF)

S2 Table Fixed-effects models predicting hours worked at main job by immigrant status and race/ethnicity in 2020 relative to 2019. Notes: *p < .05, **p < .01, ***p < .001.
(PDF)

S3 Table Fixed-effects models predicting men’s full-time employment by immigrant status, race/ethnicity and partnership in marriage or cohabitation in 2020 relative to 2019. Notes: *p < .05, **p < .01, ***p < .001.
(PDF)

S4 Table Fixed-effects models predicting men’s full-time employment by immigrant status, race/ethnicity and parenthood status in 2020 relative to 2019. Notes: *p < .05, **p < .01, ***p < .001.
(PDF)

S5 Table Fixed-effects models predicting men’s full-time employment by immigrant status, race/ethnicity and employment status of other household members in 2020 relative to 2019. Notes: *p < .05, **p < .01, ***p < .001.
(PDF)
