# Supplementary figures and images for: Unequal effects of the COVID-19 epidemic on employment: Differences by immigrant status and race/ethnicity
Source: PLoS One. 2022 Nov 15;17(11):e0277005. doi: 10.1371/journal.pone.0277005 (PMC9665404; doi:10.1371/journal.pone.0277005)

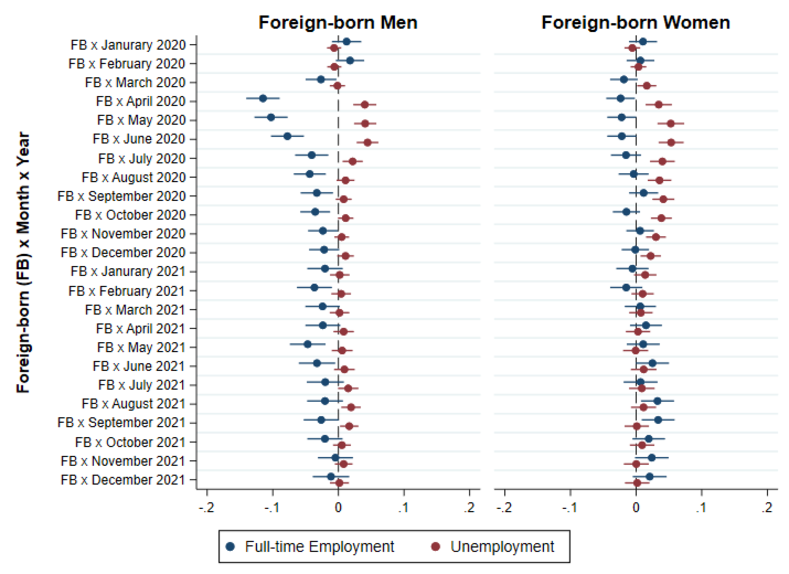

Supplement: S1 Fig — (TIF) [file pone.0277005.s001.tif]
