# Supplementary material for: Unequal effects of the COVID-19 epidemic on employment: Differences by immigrant status and race/ethnicity
Source: PLoS One. 2022 Nov 15;17(11):e0277005. doi: 10.1371/journal.pone.0277005 (PMC9665404; doi:10.1371/journal.pone.0277005)
Supplement: S1 Table — Notes: *p < .05, **p < .01, ***p < .001. (PDF) [file pone.0277005.s002.pdf]

Table S1. Robustness check: Counting full-time employed individuals not working full-time at the time of interview as full-time workers

	Full-time Employment					
	Men			Women		
	Black	Hispanic	Asian	Black	Hispanic	Asian
*Foreign-born*Month 2020*						
Foreign-born*January 2020	-0.027	0.033*	-0.004	-0.038	0.004	0.023
Foreign-born*February 2020	0.025	0.009	-0.004	-0.026	0.020	0.020
Foreign-born*March 2020	0.032	-0.020	-0.015	-0.065	-0.007	0.009
Foreign-born*April 2020	-0.155***	-0.103***	-0.023	0.010	-0.030*	0.015
Foreign-born*May 2020	-0.101*	-0.109***	-0.033	-0.062*	-0.057***	0.006
Foreign-born*June 2020	-0.132***	-0.106***	-0.058**	-0.041	-0.052***	-0.033
Foreign-born*July 2020	0.019	-0.085***	-0.039*	-0.065*	-0.015	-0.021
Foreign-born*August 2020	-0.047	-0.077***	-0.019	-0.053	-0.034*	-0.012
Foreign-born*September 2020	-0.081*	-0.043**	-0.023	-0.079*	-0.033*	-0.026
Foreign-born*October 2020	-0.041	-0.047***	-0.020	-0.072*	-0.040**	-0.039*
Foreign-born*November 2020	-0.051	-0.036**	-0.008	-0.064	-0.027	-0.009
Foreign-born*December 2020	-0.078*	-0.019	-0.020	-0.022	-0.019	0.011
*Native-born*Month 2020*						
Native-born*January 2020	-0.015	0.020	0.016	0.011	0.007	0.049*
Native-born*February 2020	-0.025	-0.004	-0.002	0.015	0.014	0.020
Native-born*March 2020	-0.011	-0.023	0.018	0.005	0.013	0.006
Native-born*April 2020	-0.031	-0.031*	-0.010	-0.005	-0.006	-0.013
Native-born*May 2020	-0.027	-0.048**	-0.019	-0.041**	-0.009	-0.036
Native-born*June 2020	-0.026	-0.067***	0.016	-0.036*	-0.014	-0.057*
Native-born*July 2020	-0.036*	-0.044**	-0.010	-0.039*	-0.013	0.009
Native-born*August 2020	-0.020	-0.026	-0.050	-0.049***	-0.003	-0.033
Native-born*September 2020	-0.032*	-0.047***	-0.038	-0.047***	-0.009	-0.033
Native-born*October 2020	-0.025	-0.043**	-0.016	-0.036**	-0.011	-0.026
Native-born*November 2020	-0.023	-0.034*	0.026	-0.038**	-0.006	-0.031
Native-born*December 2020	-0.015	-0.028	0.047*	-0.044***	-0.004	-0.040
Individual Fixed Effects	Yes	Yes	Yes	Yes	Yes	Yes
Foreign-born*Month Fixed Effects	Yes	Yes	Yes	Yes	Yes	Yes
Native-born*Month Fixed Effects	Yes	Yes	Yes	Yes	Yes	Yes
State-month-year Fixed Effects						
Industry/Occupation Fixed Effects						
Constant	0.794***	0.802***	0.813***	0.637***	0.609***	0.624***
Observations	515417	558899	498676	546407	580494	518559
Adjusted R-squared	0.724	0.688	0.719	0.755	0.757	0.769

Notes:

*p<.05, **p<.01, ***p<.001
