# Supplementary material for: Unequal effects of the COVID-19 epidemic on employment: Differences by immigrant status and race/ethnicity
Source: PLoS One. 2022 Nov 15;17(11):e0277005. doi: 10.1371/journal.pone.0277005 (PMC9665404; doi:10.1371/journal.pone.0277005)
Supplement: S2 Table — Notes: *p < .05, **p < .01, ***p < .001. (PDF) [file pone.0277005.s003.pdf]

Table S2. Fixed-effects models predicting hours worked at main job by immigrant status and race/ethnicity in 2020 relative to 2019

	Men			Women		
	Black	Hispanic	Asian	Black	Hispanic	Asian
*Foreign-born*Month 2020*						
Foreign-born*January 2020	-1.077	0.652	0.144	-0.629	1.116	0.018
Foreign-born*February 2020	0.612	1.140*	0.647	-0.051	0.668	-0.019
Foreign-born*March 2020	-0.456	0.078	-0.062	-0.032	-0.693	0.121
Foreign-born*April 2020	-4.227*	-3.274***	0.131	1.059	-2.706**	-0.254
Foreign-born*May 2020	-2.643	-3.427***	-0.925	-0.659	-4.071***	-0.801
Foreign-born*June 2020	-3.464	-2.823***	-2.138*	-2.298	-3.340***	-2.199*
Foreign-born*July 2020	-0.800	-1.640*	-0.706	-2.315	-2.239**	-1.864*
Foreign-born*August 2020	-0.776	-1.600*	-0.368	-2.387	-1.759*	-1.562
Foreign-born*September 2020	-1.938	-0.274	-0.416	-1.670	-1.293	-1.926*
Foreign-born*October 2020	-0.663	-1.738**	-0.153	-0.292	-1.849**	-1.713*
Foreign-born*November 2020	-0.685	-0.468	-0.302	-1.085	-0.460	-0.573
Foreign-born*December 2020	-1.499	-0.552	-0.753	1.797	-1.352*	0.407
*Native-born*Month 2020*						
Native-born*January 2020	-0.562	0.327	-0.371	0.826	0.421	-0.136
Native-born*February 2020	0.146	0.319	-0.471	0.926	0.577	0.909
Native-born*March 2020	-0.468	0.123	0.135	0.743	0.648	0.328
Native-born*April 2020	-0.573	-1.510*	-0.727	-0.195	-0.238	-0.372
Native-born*May 2020	-0.627	-1.267	-0.214	-0.779	-0.159	-0.618
Native-born*June 2020	0.325	-1.748*	0.425	-1.382*	-0.006	-0.033
Native-born*July 2020	-1.052	-1.610*	0.013	-2.004**	-0.849	0.683
Native-born*August 2020	-0.497	-1.673*	-0.266	-1.181	-0.765	-0.192
Native-born*September 2020	-0.171	-0.896	0.758	-0.963	-0.305	-1.990*
Native-born*October 2020	-1.112	-1.180	1.308	-1.108*	-0.653	-0.997
Native-born*November 2020	-1.015	-0.804	1.253	-0.857	-0.550	0.080
Native-born*December 2020	-0.689	-1.996**	1.442	-0.297	-0.987	-1.848*
Individual Fixed Effects	Yes	Yes	Yes	Yes	Yes	Yes
Foreign-born*Month Fixed Effects	Yes	Yes	Yes	Yes	Yes	Yes
Native-born*Month Fixed Effects	Yes	Yes	Yes	Yes	Yes	Yes
State-month-year Fixed Effects						
Industry/Occupation Fixed Effects						
Constant	40.911***	40.571***	41.157***	36.248***	36.047***	36.530***
Observations	443101	487752	434742	411833	427617	388977
Adjusted R-squared	0.568	0.553	0.557	0.610	0.611	0.617

Notes:

*p<.05, **p<.01, ***p<.001
