# Supplementary material for: Unequal effects of the COVID-19 epidemic on employment: Differences by immigrant status and race/ethnicity
Source: PLoS One. 2022 Nov 15;17(11):e0277005. doi: 10.1371/journal.pone.0277005 (PMC9665404; doi:10.1371/journal.pone.0277005)
Supplement: S3 Table — Notes: *p < .05, **p < .01, ***p < .001. (PDF) [file pone.0277005.s004.pdf]

Table S3. Fixed-effects models predicting men's full-time employment by immigrant status, race/ethnicity and partnership in marriage or cohabitation in 2020 relative to 2019

	**Men by marital status**					
	Black		Hispanic		Asian	
Married or Cohabitating?	Yes	No	Yes	No	Yes	No
*Foreign-born*Month 2020*						
Foreign-born*January 2020	-0.036	0.023	0.028	0.058	0.005	0.011
Foreign-born*February 2020	0.002	0.114	0.022	0.062	0.001	-0.033
Foreign-born*March 2020	-0.012	-0.023	-0.033	-0.010	-0.011	-0.007
Foreign-born*April 2020	-0.217***	-0.069	-0.176***	-0.120**	-0.067**	-0.027
Foreign-born*May 2020	-0.099	-0.092	-0.139***	-0.164***	-0.071**	-0.096
Foreign-born*June 2020	-0.144**	-0.149*	-0.091***	-0.135***	-0.029	-0.140**
Foreign-born*July 2020	-0.006	0.002	-0.082***	-0.056	-0.020	-0.072
Foreign-born*August 2020	-0.039	-0.112	-0.083***	-0.066	-0.031	-0.019
Foreign-born*September 2020	-0.026	0.005	-0.008	-0.072*	-0.066**	-0.041
Foreign-born*October 2020	0.018	-0.148*	-0.039*	-0.081*	-0.021	-0.010
Foreign-born*November 2020	0.021	-0.110	-0.032	-0.060	-0.013	0.035
Foreign-born*December 2020	-0.030	-0.104	-0.026	0.022	-0.043	-0.020
*Native-born*Month 2020*						
Native-born*January 2020	-0.016	0.007	0.050*	-0.004	0.027	0.024
Native-born*February 2020	-0.015	-0.005	0.033	0.025	0.023	0.015
Native-born*March 2020	-0.030	0.014	0.015	-0.029	0.024	0.026
Native-born*April 2020	-0.074**	-0.020	-0.024	-0.064*	-0.035	0.039
Native-born*May 2020	-0.072**	-0.016	-0.060**	-0.041	0.013	-0.037
Native-born*June 2020	-0.050*	-0.015	-0.053*	-0.058*	0.033	-0.030
Native-born*July 2020	-0.060*	-0.021	-0.046*	-0.071*	0.041	-0.017
Native-born*August 2020	-0.028	-0.012	-0.051*	-0.032	-0.024	0.023
Native-born*September 2020	0.007	0.027	-0.029	-0.018	-0.032	-0.013
Native-born*October 2020	-0.014	-0.041	-0.021	-0.067*	0.014	0.044
Native-born*November 2020	-0.022	-0.028	-0.027	-0.024	0.019	0.059
Native-born*December 2020	-0.028	-0.035	-0.032	-0.013	0.044	0.083
Constant	0.803***	0.603***	0.792***	0.618***	0.809***	0.624***
Observations	343984	169589	382362	174501	343975	152957
Adjusted R-squared	0.506	0.637	0.480	0.619	0.488	0.632

Notes:

*p<.05, **p<.01, ***p<.001
