# Supplementary material for: Unequal effects of the COVID-19 epidemic on employment: Differences by immigrant status and race/ethnicity
Source: PLoS One. 2022 Nov 15;17(11):e0277005. doi: 10.1371/journal.pone.0277005 (PMC9665404; doi:10.1371/journal.pone.0277005)
Supplement: S4 Table — Notes: *p < .05, **p < .01, ***p < .001. (PDF) [file pone.0277005.s005.pdf]

Table S4. Fixed-effects models predicting men's full-time employment by immigrant status, race/ethnicity and parenthood status in 2020 relative to 2019

	Men by age of child					
	Black		Hispanic		Asian	
Young children under 12 in household?	Yes	No	Yes	No	Yes	No
*Foreign-born*Month 2020*						
Foreign-born*January 2020	-0.021	-0.043	0.035	0.059	0.024	0.010
Foreign-born*February 2020	0.039	-0.084	0.025	0.033	0.003	-0.034
Foreign-born*March 2020	-0.018	0.070	-0.033	-0.015	0.002	-0.027
Foreign-born*April 2020	-0.201**	-0.209*	-0.178***	-0.161***	-0.032	-0.160**
Foreign-born*May 2020	-0.080	-0.192*	-0.109***	-0.185***	-0.024	-0.245***
Foreign-born*June 2020	-0.081	-0.209**	-0.093**	-0.118**	0.017	-0.154**
Foreign-born*July 2020	0.056	-0.126	-0.094**	-0.085*	-0.019	-0.106*
Foreign-born*August 2020	-0.022	-0.043	-0.122***	-0.055	-0.032	-0.067
Foreign-born*September 2020	0.014	-0.043	-0.035	-0.016	-0.045	-0.153**
Foreign-born*October 2020	0.006	0.005	-0.073**	-0.032	-0.038	-0.052
Foreign-born*November 2020	0.045	0.041	-0.036	-0.039	-0.044	-0.016
Foreign-born*December 2020	-0.048	-0.014	-0.039	-0.013	-0.033	-0.122**
*Native-born*Month 2020*						
Native-born*January 2020	-0.015	0.002	0.024	0.022	0.016	-0.041
Native-born*February 2020	-0.050	-0.031	0.021	0.003	0.003	0.007
Native-born*March 2020	-0.026	-0.058	0.023	0.020	0.011	0.083
Native-born*April 2020	-0.093*	-0.126**	-0.041	-0.080	-0.048	0.024
Native-born*May 2020	-0.089*	-0.096*	-0.051	-0.138**	0.012	0.008
Native-born*June 2020	-0.083*	-0.078	-0.075*	-0.042	0.062	0.102
Native-born*July 2020	-0.069	-0.068	-0.068*	-0.024	0.089	0.043
Native-born*August 2020	-0.062	-0.061	-0.052	-0.046	0.024	-0.044
Native-born*September 2020	0.009	0.029	-0.062*	-0.013	0.003	-0.028
Native-born*October 2020	-0.046	0.017	-0.054	-0.013	0.021	-0.043
Native-born*November 2020	-0.070*	0.042	-0.033	-0.014	0.085	-0.015
Native-born*December 2020	-0.004	-0.033	-0.029	-0.032	0.005	-0.091
Constant	0.813***	0.802***	0.802***	0.786***	0.822***	0.812***
Observations	154357	85674	176562	96913	155349	84365
Adjusted R-squared	0.468	0.529	0.439	0.506	0.443	0.510

Notes:

*p<.05, **p<.01, ***p<.001
