# Supplementary material for: Unequal effects of the COVID-19 epidemic on employment: Differences by immigrant status and race/ethnicity
Source: PLoS One. 2022 Nov 15;17(11):e0277005. doi: 10.1371/journal.pone.0277005 (PMC9665404; doi:10.1371/journal.pone.0277005)
Supplement: S5 Table — Notes: *p < .05, **p < .01, ***p < .001. (PDF) [file pone.0277005.s006.pdf]

Table S5. Fixed-effects models predicting men's full-time employment by immigrant status, race/ethnicity and employment status of other household members in 2020 relative to 2019

	**Men by other household member's employment**					
Any other full-time employed	Black		Hispanic		Asian	
household member?	Yes	No	Yes	No	Yes	No
*Foreign-born*Month 2020*						
Foreign-born*January 2020	0.061	0.143*	0.038	-0.01	0.031	0.047
Foreign-born*February 2020	-0.004	0.077	0.028	0.024	0.053	-0.042
Foreign-born*March 2020	-0.091	-0.013	-0.04	0.002	0.023	-0.051
Foreign-born*April 2020	-0.164*	-0.093	-0.125***	-0.169***	0.011	-0.131**
Foreign-born*May 2020	-0.044	-0.179*	-0.153***	-0.115***	-0.053	-0.153***
Foreign-born*June 2020	-0.062	-0.194*	-0.117***	-0.076*	-0.064	-0.096*
Foreign-born*July 2020	-0.077	-0.075	-0.029	-0.081*	0	-0.03
Foreign-born*August 2020	-0.043	-0.071	-0.031	-0.108***	-0.018	-0.036
Foreign-born*September 2020	0.004	0.057	0.024	-0.028	-0.066	-0.014
Foreign-born*October 2020	-0.075	0.015	-0.066*	-0.037	0.012	-0.043
Foreign-born*November 2020	-0.021	0.038	-0.065*	0.009	0.018	-0.018
Foreign-born*December 2020	-0.068	-0.147*	-0.027	0.017	0.001	-0.084**
*Native-born*Month 2020*	-0.002	0.068*	0.039	0.016	-0.031	-0.021
Native-born*January 2020	-0.016	0.015	0.019	-0.007	-0.02	0.034
Native-born*February 2020	-0.007	-0.004	-0.028	-0.034	0.104*	0.021
Native-born*March 2020	-0.064	-0.024	-0.064*	-0.034	0.006	-0.008
Native-born*April 2020	-0.072*	-0.031	-0.049	-0.064*	-0.029	0.061
Native-born*May 2020	-0.042	0.017	-0.066*	-0.082**	-0.032	0.038
Native-born*June 2020	-0.043	0	-0.052	-0.066*	-0.019	0.051
Native-born*July 2020	-0.014	-0.018	-0.042	-0.059*	0	0.012
Native-born*August 2020	0.007	0.069*	-0.038	0.017	0.014	-0.034
Native-born*September 2020	0.012	-0.04	-0.089**	-0.008	0.006	-0.014
Native-born*October 2020	0.017	-0.052	-0.034	-0.015	0.029	0.013
Native-born*November 2020	0.002	-0.060*	-0.049	-0.016	0.05	0.11
Native-born*December 2020	0.785***	0.658***	0.783***	0.683***	0.797***	0.694***
Constant	176582	182078	193027	195313	173441	175373
Observations	0.56	0.615	0.54	0.582	0.544	0.593
Adjusted R-squared	0.608	0.607	0.589	0.578	0.600	0.588

Notes:

*p<.05, **p<.01, ***p<.001
